# Biomechanical effects of semi-constrained integrated artificial discs on zygapophysial joints of implanted lumbar segments

**DOI:** 10.3892/etm.2013.1313

**Published:** 2013-09-26

**Authors:** SHENG-NAI ZHENG, QING-QIANG YAO, LI-MING WANG, WEN-HAO HU, BO WEI, YAN XU, DONG-SHENG ZHANG

**Affiliations:** 1 Department of Orthopaedic Surgery, Nanjing Medical University Nanjing Hospital, Nanjing, Jiangsu 210029, P.R. China; 2 Biomechanical Laboratory, Shanghai University, Shanghai 200444, P.R. China

**Keywords:** lumbar vertebrae, zygapophysial joints, artificial disc, semi-constrained disc, biomechanics

## Abstract

This study aimed to optimize the design and application of semi-constrained integrated artificial discs (SIADs) using a finite element (FE) analysis following implantation, wherein the zygapophysial joints of the segment were biomechanically reconstructed. An FE model of the L4–L5 segment was constructed. Variations in the stresses on the discs and zygapophysial joints were observed during 5° anteflexion, 5° extension and 5° rotation under the 400-N applied axial load. Stresses and load translation analyses of the discs and zygapophysial joints were conducted during anteflexion, extension and rotation under the 400-N applied axial load. Following implantation of the lumbar segments, the stresses on the SIAD zygapophysial joints were not significantly different from those of physiological discs during anteflexion, and these were both marginally greater compared with those of non-constrained artificial discs (NADs). During extension, the increase in the stress on the SIAD zygapophysial joints was less than that on NAD zygapophysial joints. Stresses on the NAD zygapophysial joints were higher than those on SIAD and physiological discs during rotation. The stress on the SIAD zygapophysial joints was not significantly different from that on physiological discs during rotation. For SIADs and NADs, the stresses on the zygapophysial joints and the displacements of the discs were greater compared with those of the physiological discs during extension. The SIADs affected the variations in the stresses on the implanted segment more than the NADs, and the SIADs protected the zygapophysial joints of the implanted segment to a higher degree than the NADs.

## Introduction

Chronic lumbar pain is mainly caused by lumbar discogenic pain that originates from lumbar spondylosis. Lumbar spondylosis manifests as mechanical back pain with instability of the lumbar spine and regression of zygapophysial joints, which may develop into lumbar pain and neurogenic intermittent claudication (1,2), caused by lumbar spinal stenosis. Conservative treatments for severe lumbar spondylosis tend to be ineffective and surgery is currently the main treatment method. In addition to bone grafting and internal fixation, the surgery includes removal of the nucleus gelatinosus and/or a laminectomy for decompression. However, several studies have indicated that fusion procedures do not improve the outcome of lumbar spondylosis, even with a success rate of 98% (3). Furthermore, the analgesic period following fusion procedures that include internal fixation is shorter than the surgery itself (excluding the internal fixation) (3). The discrepancy mainly occurs due to the surgery destroying the biomechanical environment of the lumbar motion segment, particularly those that include rigid internal fixation, which prevents the load transfer from conforming to physiological conditions. This ultimately accelerates the degeneration of adjacent segment facets, loosens the internal fixation and results in fatigue fractures. Consequently, lumbar non-fusion fixation has gained increasing attention in previous years. Among numerous non-fusion fixation procedures, artificial lumbar disc replacement is regarded as a promising area of development. Its theoretical advantages include removal of the source of discogenic lumbar pain, allowance for the motion of an implanted segment, restoration of intervertebral disc height and a shorter rehabilitation time compared with that of fusion operations. At present, the predominant design of artificial lumbar disc involves reconstruction as ball and socket joints, which allow rotation of the intervertebral disc segments, such as with Prodisc-L™ (Synthes Spine, Inc., Paoli, PA, USA), which employs a polyethylene nucleus pulposus and metal soleplate (4). Additionally, Activ-L^®^ (B. Braun/Aesculap, Tuttlingen, Germany) allows rotation and retains backward motion (5); and Mobidisc^®^ (LDR Médical, Troyes, France) is capable of movement in all directions in the plane parallel to the physiological soleplate (6). However, with increasing application, the clinical effects of artificial lumbar disc replacement fail to meet the theoretical standards. Related studies have demonstrated that this failure is due to regression of the zygapophysial joints, an impractical intervertebral opening height and an undesirable prosthesis position. Rohlmann *et al*(7) identified that the occurrence of lower back pain following artificial lumbar disc replacement is associated with increased stress on the zygapophysial joints. Additionally, Kim *et al*(8) indicated that following artificial lumbar disc replacement, segment extension activity increases significantly during extension, which directly increases the stress on zygapophysial joints compared with the direct stress on the zygapophysial joints of adjacent segments. Subsequently, the increased stress causes regression of the zygapophysial joints and ultimately results in lumbar regression.

The current design of non-constrained artificial discs (NADs) may be improved by constructing semi-constrained disc prostheses, wherein a fiber structure is added to simulate the fiber-connecting properties of the physiological disc and to constrain its activity. This modification may decrease the stress on the zygapophysial joints and improve the clinical therapeutic effects of lumbar disc replacement (5). The present study investigated a newly designed semi-constrained integrated artificial disc (SIAD; Weigao Orthopaedic Device Co., Ltd., Weihai, China) with a titanium plate in its framework, polyethylene glycol terephthalate elastic ligaments that simulate the annulus fibrosus of the physiological discs and a polyetheretherketone (PEEK) core that simulates the nucleus pulposus. This study compared the effects of physiological discs, SIADs and NADs on the stresses on zygapophysial joints and the load translation of the implanted segments, using finite element (FE) analysis. Furthermore, the rationality of using the artificial disc prosthesis for the treatment of chronic lumbar pain, was evaluated.

## Materials and methods

### FE model

**Figure 1:**
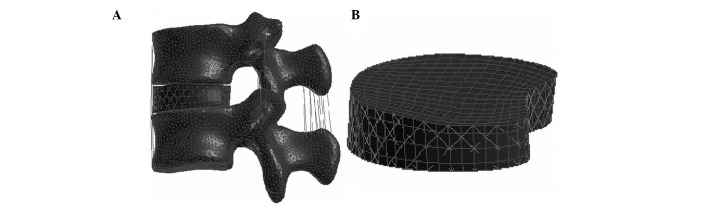
(A) Constructed L4–L5 motion segment finite element model and (B) physiological disc.

A 21-year-old male volunteer (height, 175 cm) was selected as the simulation subject. The L4–L5 spinal segment of the patient was allowed a 0.75 mm stratum depth. The data required for constructing the FE model of the lumbar L4–L5 segment was obtained through continuous computed tomography (CT) scanning. The lumbar FE model was constructed after converting data from the CT scan into 3D data using Mimics software (version 10.0, Materialise Inc., Leuven, Belgium) and Patran preprocessing software (MSC.Software Corp., Surrey, United Kingdom; Fig. 1). The centrum consisted of external cortical bone and internal cancellous bone. The cortical bone was set at 1.0 mm and the different sections were connected and simulated as a solid unit, which was then simplified into continuous, even and isotropic bone structures. The thickness of the soleplate was 1.0 mm and the interval between the zygapophysial joints was 1.0 mm. In addition, the articular cartilage surface was simulated using an area unit with a thickness of 1.0 mm. The two contact surfaces had no friction, moved relatively at a 0.6-mm distance and were simulated by a nonlinear link unit. The intervertebral disc consisted of an annulus fibrosus and a nucleus pulposus. The annulus fibrosus was simulated as a ring of stroma embedded with three layers of collagen fiber that intersected the horizontal plane of the intervertebral disc at a ±30° included angle. A linear elastic material that only accepts tensile stress was used and simulated a link linear unit. Moreover, the stroma of the annulus fibrosus was simulated as a solid unit, with an elastic modulus of 4.2. The ligament structure such as the spinal ligaments (anterior longitudinal, posterior longitudinal, inter- and supraspinal and intertransverse ligaments, ligamentum flavum and zygapophysial joint ligaments), the fibers and capsula articularis were simulated as link units using a linear elastic material that only accepts tensile stress. The elastic moduli and the Poisson’s ratio of the different parts of the FE model were in accordance with the literature (9–11) (Table I). This study was conducted in accordance with the Declaration of Helsinki with approval from the ethics committee of Nanjing Medical University Hospital (Nanjing, China). Written informed consent was obtained from the participant.

### SIAD FE model construction

**Figure 2:**
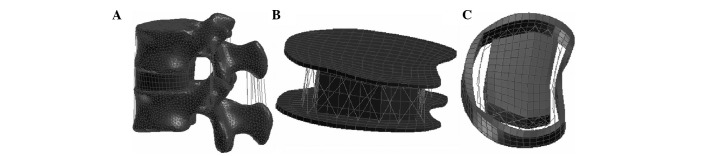
Semi-constrained integrated artificial disc (SIAD; fiber disc prosthesis). (A) L4–L5 motion segment following SIAD replacement; (B) SIAD, from which the outer surrounding has been removed and (C) SIAD (perspective drawing) of which the soleplate has been removed.

The SIAD artificial disc was integrated as an artificial disc prosthesis. The elastic modulus of its titanium soleplate was 77,000 MPa and its nucleus pulposus consisted of PEEK with an elastic modulus of 3,600 MPa. The polyethylene glycol terephthalate elastic ligament that enveloped the PEEK nucleus pulposus, was woven into the soleplate to form a ±30° included angle with the horizontal plane of the intervertebral disc. The annulus fibrosus of the physiological disc was simulated using a linear elastic material that only accepts tensile stress and a nonlinear link unit was used for simulation. Furthermore, the upper and lower soleplates were fixed with a vertebral soleplate. The nucleus pulposus and the upper and lower soleplates were set as non-friction contact surfaces that moved relatively 0.6 mm apart (Fig. 2).

### NAD FE model construction

**Figure 3:**
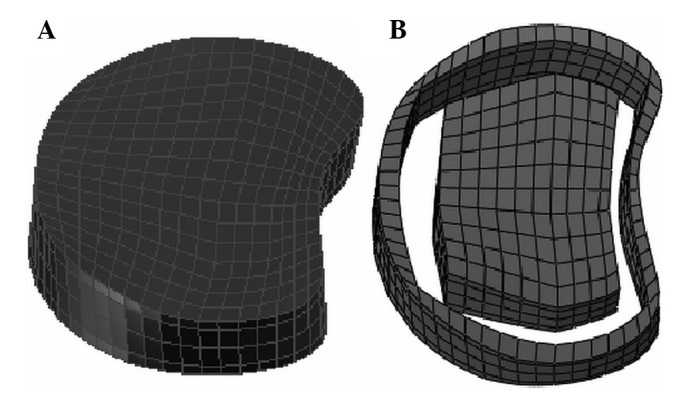
Non-constrained artificial disc (NAD) prosthesis. (A) NAD prosthesis and (B) NAD perspective drawing with the soleplate removed.

The NAD was integrated as the prosthesis with the elastic fibers removed. The elastic modulus of its titanium soleplate was 77,000 MPa and its nucleus pulposus consisted of PEEK, which was divided into upper and lower sections by a horizontal plane. The elastic modulus of the PEEK was 3,600 MPa. The different sections of the model were fixed with an adjacent titanium soleplate and the two sections were set as non-friction contact surfaces that moved relatively 0.6 mm apart (Fig. 3).

### Model verification

Normal neutral stress on the lower lumbar segment was simulated to calibrate the FE model to enable it to be compared with other lumbar FE models. Up to two-thirds of a typical human weight (~40 kg or 400 N of a 60-kg subject) was divided into four Von Mises stresses, each at 100 N. Each stress was used as a node load applied to the anterior and posterior of the vertebral joints equidistant from the L4 vertebral rotation axis by 19 steps. Von Mises stress is an equivalent stress based on shear strain energy, which is calculated using the following formula: {[(a1−a2)^2^ + (a2−a3)^2^ + (a3−a1)^2^]/2}^1/2^, where a1, a2 and a3 represent the first, second and third major stresses, respectively (9). The degrees of freedom of the structures on the lower surface of the L5 vertebra, which were considered static, were set to zero. The results of the model were compared with those of the lumbar FE models reported by Goto *et al*(10) and Grant *et al*(11).

### Stress loading

Stress loading was subdivided into two programs. The Patran preprocessing software (Dassault Systemes Simulia Corp., Providence, RI, USA) was used to construct the lumbar FE model and the Abaqus FE analysis software was used to simulate the constraints and loadings.

### Working condition stress analysis

**Figure 4:**
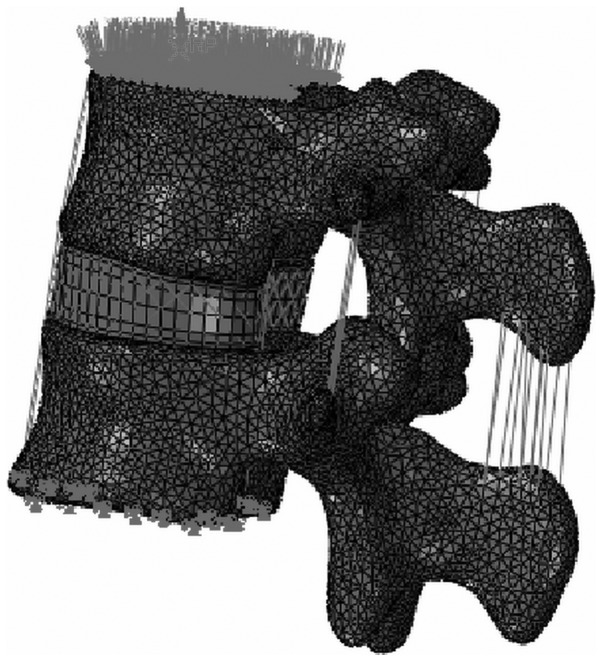
Stress application following the fixation of all node translations of the lower vertebra (L5).

A 400-N downward force was applied axially on the upper surface of the thoracic vertebra to simulate gravity on the upper body at a neutral position. From the neutral position, 5° anteflexion, 5° extension and 5° rotation were then simulated by applying a 10-Nm moment. Subsequently, stress on the zygapophysial joints was observed during 5° anteflexion, 5° extension and 5° rotation. According to the calculations, the degrees of freedom of all node translations of the L5 lumbar bottom were set to zero. A tie constraint was used between the soleplate and the disc to ensure that they did not separate (Fig. 4).

### Statistical analysis

Statistical analysis was performed using SPSS 13.0 statistical software (SPSS, Chicago, IL, USA). P<0.05 was considered to indicate a statistically significant result.

## Results

### General data

**Figure 5:**
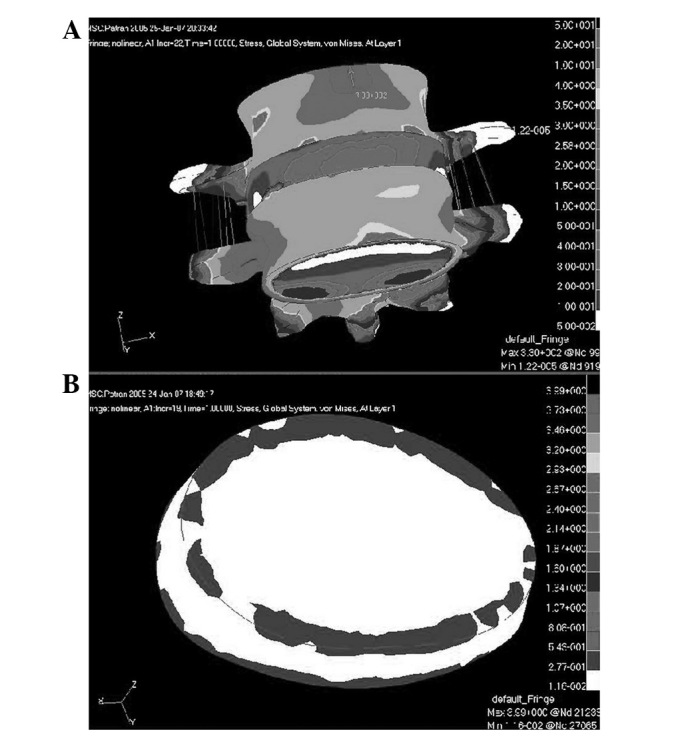
(A) Verification of the 400-N applied axial stress model and (B) lower lumbar intervertebral disc distribution of the 400-N applied axial stress.

An FE model of the L4–L5 motion segment was constructed. The model included 34,123 nodes and 162,858 units, which contained 161,679 tetrahedral centrum elements, 1,053 hexahedral disc and soleplate elements, and 126 ligament link units, capsula articularis and fibers. In the neutral position, a 400-N axial stress and a 10-Nm moment were applied to simulate loading. Under the simulated anteflexion, extension and rotation, the stresses and distribution of all units in the motion segment were consistent with the results of the lumbar FE models reported by Goto *et al*(10) and Yamamoto *et al*(11) (Fig. 5).

### Working condition stress analysis

**Figure 6:**
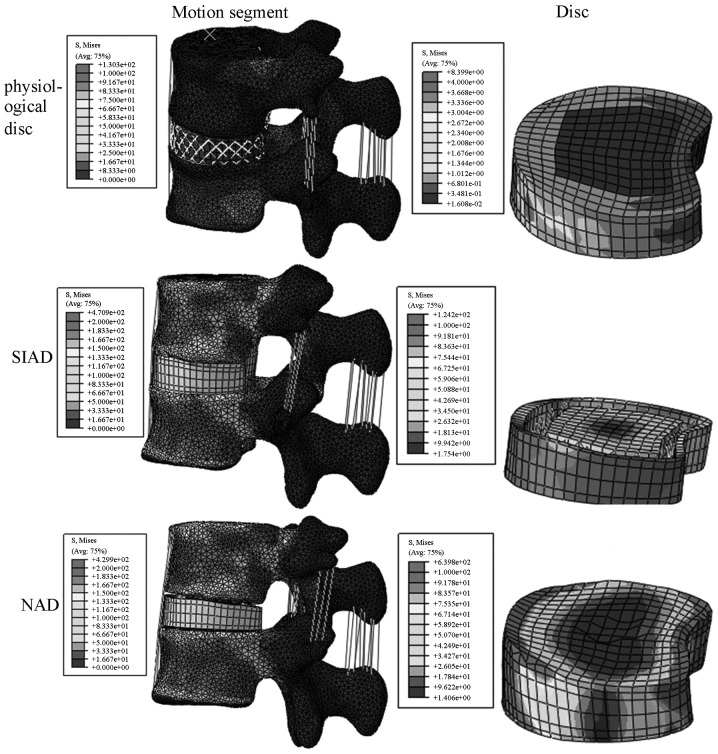
Stress distribution of 400-N applied axial stresses and right 5° rotation.

**Figure 7:**
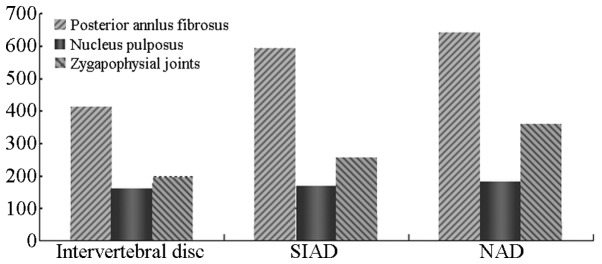
Analysis of the 400-N applied axial stress and the 5° extension moment.

**Figure 8:**
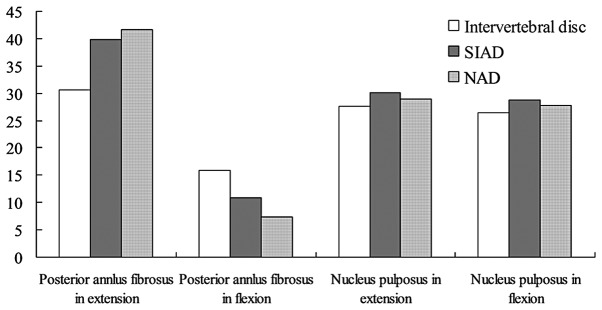
Analysis of the 400-N applied axial stress and the 10 Nm moment during anteflexion and extension.

In anteflexion, the stresses on the zygapophysial joints with physiological discs and those following implantation of the SIAD demonstrated no significant differences, and were slightly greater than those of NAD joints. During extension, the stresses on the SIAD zygapophysial joints with SIAD and NAD transplants were greater than those with physiological discs. However, the increase in SIAD zygapophysial joint stresses was lower compared with that of the NAD. During rotation, the stresses on the zygapophysial joints and on the physiological discs were not significantly different. The stresses on the NAD zygapophysial joints were significantly greater compared with those on the joints of the other two types during rotation (Tables II–V and Figs. 6–8).

The stresses on the SIAD zygapophysial joints during anteflexion were not significantly different compared with those on the zygapophysial joints of physiological discs. The zygapophysial joint stresses and displacements of the SIAD and NAD following implantation were greater than those of physiological discs during extension (Fig. 8); however, the increase in the stresses and segment activity of the SIAD zygapophysial joints was lower than that of the NAD joints. The stresses on the SIAD zygapophysial joint and those on the physiological discs during rotation were not significantly different; however, the stresses on the NAD zygapophysial joints were significantly greater than those in the joints of the other two types.

## Discussion

Lumbar degenerative disease (LDD) is one of the most common spinal diseases treated by surgery, affecting 80% of the world’s population according to Hult statistics (12). The National Center for Health Statistics of the USA reported that the most common factor for the limitation of movement among people <45 years old is lumbar pain syndrome caused by LDD. The annual treatment cost for LDD has increased to millions of dollars, excluding losses from absenteeism (13).

Treatment for chronic discogenic lumbar pain includes artificial disc replacement, which maintains the activity of the lumbar motion segment unlike traditional fusion operations (14). Therefore, artificial disc replacement is of increasing interest and FE analyses and clinical application studies of artificial discs are increasing. Artificial disc replacement is an important area for the development of treatments for lumbar spondylosis due to the following (15–17): i) it completely restores diseased intervertebral disc tissue and eliminates mechanical back pain caused by intervertebral disc disease; ii) it relieves the compression stimuli of degenerative intervertebral discs on the spinal nerve and the nerve root, and effectively releases the symptoms of nerve compression; iii) it allows the activity of the operated segment to be maintained and reduces the loss of lumbar activity following the surgical procedure; and iv) it minimally affects the lumbar biomechanical environment following implantation, with a lower incidence rate of degeneration of the adjacent segments compared with that of traditional fusion operations. Therefore, artificial disc replacement may be applied in multisegment replacements (18,19).

Various artificial disc prostheses have been designed (4,20–24). Four major artificial disc prostheses are used in the USA: Charité (DePuy Spine Inc., Raynham, MA, USA) (20) and Prodisc (Synthes Spine, Paoli, PA, USA) (4), which are metal-polythene prostheses that underwent clinical trials and registration in the USA; and Maverick (Medtronic Sofamor Danek Inc., Memphis, TN, USA) (25) and FlexiCore (Stryker Spine, Allendale, NJ, USA) (26,27), which are metal-metal (Co-Cr) interface prostheses. Artificial discs are currently classified as three-component prostheses, two-component prostheses and integrated prostheses. Regardless of the various architectural designs, the clinical effects of artificial disc replacement are considered unsatisfactory (28). For example, the two-component prostheses (Prodisc) perform the loading function of the upper and lower soleplates, respectively. They form a self-constrained articular facet, but lose the stretching-constraining function of physiological discs during lumbar twisting and have no load buffering function. The three-component prostheses (Charité) consist of upper and lower soleplates and an elastic core that forms the articular facet of the prosthesis following composition (29). However, three-component prostheses have comparatively greater activity and do not feature disc-constrained motion, which subsequently causes strain on the rear zygapophysial joints that results in unsatisfactory long-term clinical effects. The therapeutic effects of this type of prosthesis do not meet theoretical expectations; therefore, disc fiber-connecting mechanisms have gained increasing attention. With this mechanism, a disc has a deformable fiber cartilage joint in place of a movable vertebral synovial joint. A multi-cartilage joint inhibits the range of activity and does not achieve the buckling stress-strain curve of the physiological discs (Fig. 1). Therefore, all the described prostheses result in biomechanical changes in the implanted segments. A key reason for the unsatisfactory clinical effects is the secondary degeneration of the implanted segments due to accelerated degeneration of zygapophysial joints following the surgery and secondary spinal stenosis. Therefore, development of an integrated prosthesis that matches the disc fiber-connecting function has become a topic of great interest in artificial disc development (21–24).

McNally *et al*(30) anchored a multi-hardness elastic nucleus composed of polycarbonates in various degrees on the upper and lower titanium plates. This modification allowed the implanted segments to rotate by continuously centering on the physiological rotation axis. Keels on the upper and lower titanium plates are associated with the soleplate and facilitate bone fusion. However, in this design, which is named Physio-L. the absence of separate elastic components and its increased technical complexity decrease the reliability of the system, as it lacks the buckling stress-strain curve of the annulus fibrosus of the physiological discs. Its elastic core has weak points and its upper and lower titanium plates, which do not contain elastic components, readily cause structural failure under shearing loads. The Cadisc™-L prosthesis designed by Ranier Technology Ltd. (Cambridge, UK) (31) is made of polycarbonate polyurethane materials that are integrated into the structure. The buckling stress-strain curve of the physiological discs was incorporated; however, metals were not used in the upper and lower plates, due to the shaping and workmanship required, which subsequently may affect stability as it hinders bone fusion with the prosthesis. Furthermore, it is not possible to examine the prosthesis by X-ray, which is inconvenient during operative installation and postoperative follow-up (32,33).

In the present study, the SIAD structure had an integrated semi-constrained artificial disc, with a soleplate composed of titanium and polyethylene glycol terephthalate. An elastic ligament was anchored onto the soleplate to simulate the annulus fibrosus and a PEEK core was used as the elastic nucleus pulposus. Theoretically, the SIAD simulates the connection of physiological disc fibers and limits rotation, which in turn decreases the stresses on the zygapophysial joints and improves the clinical effects of disc replacement.

Further studies are required to verify the aforementioned hypothesis. At present, common mechanical research methods include animal experiments, physical experiments, *in vitro* (cadaver) experiments and computer simulations. The animals used in previous animal experiments were non-erect and were markedly different in structure and function compared with humans. Physical experiments have limited value due to the lack of *in vivo* structural characteristics. However, the cost of *in vitro* experiments is high and finding cadavers is difficult. Moreover, *in vitro* experimental conditions greatly differ from *in vivo* conditions (34). Notably, Thresher and Farah were among the first to use the FE method (FEM) in the medical field. Since 1973, the FEM has become an effective mathematical tool in human biomechanical studies (35). Therefore, the present study attempted to construct an FE model of an early-stage degenerative lumbar motion segment to simulate the biomechanical changes. The suitability of constructing an L4–L5 segment FE model was demonstrated by calibrating with the Goto *et al*(10) and Yamamoto *et al*(11) standard lumbar models.

Following SIAD implantation, the increase in pressure on the zygapophysial joint segments was only 79% that with NAD implantation under a 400-N applied axial load and 10-Nm moment during extension, whereas the translation was at 65% with NAD. Furthermore, the load on the annulus fibrosus of the SIAD increased, which demonstrated that the semi-constrained prostheses protected the zygapophysial joints of implanted segments during extension (153.3 MPa vs. 193.9 MPa). Under this load, the corresponding degree of translational stress on the zygapophysial joints following SIAD and physiological disc implantation was higher than that for the NAD. Therefore, the SIAD design affects the segment functional spinal unit less than the NAD does. Under a 400-N applied axial load and a 10-Nm right rotation moment, the stresses on the segments of the zygapophysial joints with SIAD and NAD implants increased compared with those on joints with physiological discs. However, the increases in the stresses and the translation on the zygapophysial joints of the NAD were higher compared with those of the SIAD. Therefore, the semi-constrained design has similar mechanical characteristics to physiological discs and helps to protect the zygapophysial joints. Under an applied axial load of 400 N with a 5° extension or 5° rotation moment, the stresses on the SIAD zygapophysial joints were lower than those on the NAD. Therefore, the semi-constrained design of the SIAD buffers the stresses on the segment motion, which subsequently protects the zygapophysial joints.

In the present study, the incision and suture of the anterior and posterior longitudinal ligaments, as well as the preservation of the structure of the *Annulus fibrosus*, were not considered in the calculations of the working conditions. All working conditions were simplified as in disc replacement. This study demonstrated that based on the FE analysis, the SIAD protected the zygapophysial joints of the implanted segment. Therefore, application of the design may improve the clinical therapeutic effects of artificial discs.

## Figures and Tables

**Table I:** Elastic modulus, Poisson ratio and sectional area of different structures.

Structure	Elastic modulus (MPa)	Poisson ratio	Sectional area (mm^2^)
Titanium plate	77000.0	0.3000	-
PEEK	3600.0	0.3000	-
Cortical bone	12000.0	0.3000	-
Cancellous bone	100.0	0.2000	-
Articular cartilage	10.0	0.4000	-
Posterior part of the vertebral body	3500.0	0.2500	-
Lamina terminalis	1000.0	0.4000	-
Matrix of fibrous ring of intervertebral disc	4.2	0.4500	-
Nucleus pulposus intervertebral disc	0.2	0.4999	-
Fiber of fibrous ring	450.0	0.3000	0.15
Anterior longitudinal ligament	20.0	0.3000	38.00
Posterior longitudinal ligament	70.0	0.3000	20.00
Ligament flavum	50.0	0.3000	60.00
Interspinous ligament	28.0	0.3000	35.50
Supraspinous ligament	28.0	0.3000	35.50
Intertransverse ligament	50.0	0.3000	10.00
Articular capsule	100.0	0.3000	40.00

PEEK, polyetheretherketone.

**Table II:** Stress analysis under 400-N applied axial stress at 5° flexion, 5° extension and neutral positions.

	Posterior ring of intervertebral disc stress (MPa)			Nucleus pulposus stress (MPa)			Zygapophysial joint stress (MPa)		
			
Disc type	Extension	Neutral	Flexion	Extension	Neutral	Flexion	Extension	Neutral	Flexion
Physiological disc	410.9	42.7	21.4	159.8	151.2	155.2	193.1	114.5	63.6
SIAD	592.4	44.8	19.8	169.3	170.7	151.6	253.8	118.3	62.9
NAD	639.8	59.9	17.5	181.1	178.8	149.6	357.8	113.4	43.9

SIAD, semi-constrained integrated artificial disc and NAD, non-constrained artificial disc.

**Table III:** 400-N applied axial stress and stress analysis at right 5° rotation position.

Disc type	Posterior ring of intervertebral disc stress (MPa)	Nucleus pulposus stress (MPa)	Lateral ring of intervertebral disc stress (MPa)	Zygapophysial joint stress (MPa)
Physiological disc	46.6	154.5	83.5	79.9
SIAD	42.8	168.1	54.8	91.5
NAD	53.7	175.7	24.6	117.8

SIAD, semi-constrained integrated artificial disc; NAD, non-constrained artificial disc.

**Table IV:** Stress analysis under 400 N applied axial stress and a 10 Nm moment at flexion and extension positions.

	Posterior ring of intervertebral disc stress (MPa)		Nucleus pulposus stress (MPa)		Zygapophysial joint stress (MPa)	
			
Disc type	Extension	Flexion	Extension	Flexion	Extension	Flexion
Physiological disc	30.4	15.9	27.4	26.3	141.4	73.2
SIAD	39.8	10.7	29.9	28.6	153.3	51.5
NAD	41.6	7.4	28.7	27.6	193.9	50.9

SIAD, semi-constrained integrated artificial discs; NAD, non-constrained artificial disc.

**Table V:** Displacement analysis of 400-N applied axial stress and 10 Nm moment at flexion and extension positions.

	Displacement of anterior ring of intervertebral disc (mm)			Displacement of posterior ring of intervertebral disc (mm)			Average displacement of zygapophysial joint (mm)		
			
Disc type	Extension	Flexion	Rotation	Extension	Flexion	Rotation	Extension	Flexion	Rotation
Physiological disc	1.0	-	0.5	-	1.0	0.2	0.4	1.2	0.2
SIAD	1.4	-	0.7	-	1.3	0.3	0.7	1.5	0.3
NAD	2.0	-	1.3	-	2.0	0.6	1.2	2.2	0.5

SIAD, semi-constrained integrated artificial disc; NAD, non-constrained artificial disc.
